# Barriers and Facilitators in Implementing a Telemonitoring Application for Patients With Chronic Kidney Disease and Health Professionals: Ancillary Implementation Study of the NeLLY (New Health e-Link in the Lyon Region) Stepped-Wedge Randomized Controlled Trial

**DOI:** 10.2196/50014

**Published:** 2025-01-22

**Authors:** Marion Delvallée, Abdallah Guerraoui, Lucas Tchetgnia, Jean-Pierre Grangier, Nassira Amamra, Anne-Laure Camarroque, Julie Haesebaert, Agnès Caillette-Beaudoin

**Affiliations:** 1 Research on Healthcare Performance RESHAPE, INSERM U1290 Université Claude Bernard Lyon 1 Lyon France; 2 Calydial Vienne France; 3 Collectif en Sciences Sociales Appliquées Paris France; 4 Université Bourgogne Europe Laboratoire Interdisciplinaire de Recherches - Sociétés, Sensibilités, Soin Dijon France; 5 Service Recherche et Epidémiologie Cliniques Pôle de Sante Publique Hospices Civils de Lyon Lyon France

**Keywords:** telehealth, telemonitoring, chronic kidney disease, implementation, Consolidated Framework for Implementation Research, Technology Acceptance Model

## Abstract

**Background:**

The use of telemonitoring to manage renal function in patients with chronic kidney disease (CKD) is recommended by health authorities. However, despite these recommendations, the adoption of telemonitoring by both health care professionals and patients faces numerous challenges.

**Objective:**

This study aims to identify barriers and facilitators in the implementation of a telemonitoring program for patients with CKD, as perceived by health care professionals and patients, and to explore factors associated with the adoption of the program. This study serves as a process evaluation conducted alongside the cost-effectiveness NeLLY (New Health e-Link in the Lyon Region) trial.

**Methods:**

A mixed methods approach combining a quantitative questionnaire and semistructured interviews was conducted among nurses, nephrologists, and patients with stages 3 and 4 CKD across 10 renal care centers in France that have implemented telemonitoring. The Technology Acceptance Model (TAM) and the Consolidated Framework for Implementation Research (CFIR) were used to design the questionnaires and interview guides. The dimensions investigated included ease of use, perceived usefulness, and intention to use (TAM), as well as characteristics of the intervention, local and general context, individual factors, and processes (CFIR). The adoption of telemonitoring was assessed based on the frequency with which patients connected to the telemonitoring device. Determinants of telemonitoring use were analyzed using nonparametric tests, specifically the Wilcoxon-Mann-Whitney and Kruskal-Wallis tests. Thematic analysis was conducted on the transcriptions of semistructured interviews. Both quantitative and qualitative results, including data from patients and professionals, were integrated to provide a comprehensive understanding of the factors associated with the use of remote monitoring in CKD.

**Results:**

A total of 42 professionals and 128 patients with CKD responded to our questionnaire. Among these, 11 professionals and 13 patients participated in interviews. Nurses, who were responsible for patient follow-up, regularly used telemonitoring (8/13, 62%, at least once a month), while nephrologists, who were responsible for prescribing it, were primarily occasional users (5/8, 63%, using it less than once a month). Among professionals, the main obstacles identified were the heavy workload generated by telemonitoring, lack of training, and insufficient support for nurses. Among the 128 patients, 46 (35.9%) reported using the application at least once a week. The main barriers for patients were issues related to computer use, as well as the lack of feedback and communication with health care professionals. The main facilitators identified by both professionals and patients for using telemonitoring were the empowerment of patients in managing their health and the reduction of the burden associated with CKD.

**Conclusions:**

Improving adherence to telemonitoring in the context of CKD requires collaborative efforts from both professionals and patients. Our results provide insights that can inform the design of effective, theory-driven interventions aimed at improving telemonitoring adoption and usage.

## Introduction

Chronic kidney disease (CKD) is a generally asymptomatic condition characterized by a progressive decline in kidney function. The prevalence of CKD is approximately 11% in high-income countries [1,2]. The primary challenge for health professionals is to preserve patients’ renal function for as long as possible to delay progression to stage 5, where kidney function must be compensated by dialysis or transplantation. To achieve this, it is necessary to closely monitor patients’ health status—such as blood pressure, creatinine levels, or weight [3]—from the onset of the disease [4]. This adds to the burden of the disease for patients and may lead to challenges with adherence and engagement in their care. In the context of CKD, telemonitoring, which involves remotely tracking a patient’s health status through regular collection of health data, has been internationally recommended [5] and in France “to slow the progression of the disease by setting up early detection of kidney disease in patients at risk and appropriate therapeutic management” [6]. Indeed, in patients with CKD, telemonitoring has been shown to improve data sharing between patients and health professionals, enhance patient autonomy (eg, blood pressure control) [7], facilitate care coordination [5], and support the monitoring of patients in remote areas [8]. Additionally, some studies have reported better compliance in these patients [9-12].

Although telemonitoring is recommended for CKD in many countries to adapt patient care [13], several studies have raised concerns about its feasibility [14,15]. Bonner et al [16] highlighted that factors such as health literacy, sociodemographic characteristics, and ease of use of technology could influence telehealth adoption in patients with CKD. In a recent review, Jacob et al [17] classified the factors influencing telehealth adoption by professionals into 3 categories. These categories include (1) technical and material factors (eg, ease of use, user experience), (2) social and personal factors (eg, personal characteristics), and (3) organizational and policy factors (eg, workflow-related and patient-related considerations). Another review by O’Connor et al [18] explained patient engagement factors in telehealth. These factors were divided into 4 categories: (1) personal agency and motivation, (2) personal life and values, (3) engagement and recruitment approaches, and (4) quality of the digital health intervention. Several theoretical frameworks have been proposed to organize and group these factors into domains to guide telehealth implementation [19].

Studies have shown that telehealth implementation impacts patients and professionals both positively and negatively [17,19-23]. However, these impacts may vary depending on cultural and organizational contexts. Currently, no data are available in France regarding the acceptability and adoption of telemonitoring for the follow-up of patients with CKD. A French national multicenter stepped-wedge randomized controlled trial (SW-RCT) (NeLLY [New Health e-Link in the Lyon Region] Trial NCT03348839) is ongoing to evaluate the cost-effectiveness of a program that combines multiprofessional telemonitoring with support for a personalized care pathway for patients with severe predialysis CKD. In the NeLLY program, telemonitoring is delivered through the apTeleCare application (Multimedia Appendix 1). This application enables the planning and monitoring of scheduled or unscheduled activities, as well as the management of alerts triggered when patients enter concerning health data. Patients and health professionals can access the NeLLY program when their center is in the intervention phase of the SW-RCT.

The aim of our study was to identify the barriers and facilitators to implementing the NeLLY telemonitoring program by gathering insights from health professionals and patients with CKD participating in the NeLLY trial.

## Methods

### Study Design

We conducted a mixed methods implementation survey, combining quantitative questionnaires and semistructured interviews, following an explanatory sequential design [24]. This survey was conducted alongside the NeLLY national multicenter SW-RCT. The aim of our implementation survey was to assess the use of remote monitoring by patients and professionals in the units participating in the trial. Our survey was conducted in the 10 centers (out of a total of 15 participating centers) that were in the intervention phase at the time of the survey.

### Ethics Approval

The Institutional Review Board CPP OUEST II-Angers approved this study, project no. 2017/37 (2017-A00091-52). The reporting of the results is guided by the Checklist for Reporting Results of Internet E-Surveys (CHERRIES) guidelines [25]. All data were anonymized with a unique anonymity number. No compensation was provided to participants for the study.

### Theoretical Framework

We based the design and conduct of our study on 2 complementary theoretical frameworks: the Technology Acceptance Model (TAM), specific to telemedicine adoption, supplemented by additional domains from the general implementation framework—the Consolidated Framework for Implementation Research (CFIR)—to achieve a comprehensive understanding of the factors shaping implementation.

The TAM focuses on understanding why users accept or reject technology and how acceptance can be improved [26]. It is based on 2 key principles: perceived usefulness and perceived ease of use. These concepts shape users’ attitudes toward technology, which, in turn, influence behavioral intention. This framework can help identify factors for optimizing the implementation of telehealth.

The CFIR examines 5 areas: intervention characteristics, outer settings, inner settings, characteristics of individuals, and the implementation process [27]. It helps in understanding implementation and can be used to guide formative assessments. This framework has been widely applied in studies to explore factors influencing the implementation of telehealth [28].

TAM and CFIR share common domains, but TAM includes domains that are more specific to telemonitoring. CFIR, by contrast, incorporates additional domains related to intervention characteristics, outer settings, inner settings, individuals, and processes, which we integrated to develop our framework.

### Participants

Our study targeted 2 populations: patients and health professionals participating in the NeLLY trial at centers in the intervention phase during our study.

The patients included in the NeLLY trial were those with CKD at stage 4 or a glomerular filtration rate between 30 and 37 ml/minute, as determined based on the nephrologist’s assessment. Eligible patients also had at least one comorbidity (cardiovascular disease, diabetes, or both) and access to an internet connection at home. All patients enrolled in the intervention phase of PRME (Medical and Economic Research Program) NeLLY at the start of our survey (March 2021) were invited to participate and complete the questionnaire. A total of 305 patients were invited to the study, and all voluntary respondents were included in the survey.

In each center, health professionals—including nephrologists and nurses—who were involved in recruiting patients for the NeLLY trial or monitoring patients at the time of the study were eligible to participate. A total of 103 eligible professionals were contacted, and all voluntary respondents were included in the survey.

### Development of Questionnaires and Interview Guides

Questionnaires and interview guides were developed based on the 3 categories of factors described by Jacob et al [17] and informed by the CFIR and TAM frameworks [26-29]. The first question of the survey addressed participants’ nonopposition to study participation. The patient questionnaire comprised 29 items divided into 3 groups: sociodemographic factors (4 items), technological and material factors (18 items), and social and personal factors (5 items). The professionals’ questionnaire consisted of 45 items divided into 4 sections: sociodemographic factors (8 items), technological and material factors (14 items), social and personal factors (6 items), and organizational and policy factors (15 items). A table detailing the questions by domains of the theoretical framework is available in Multimedia Appendix 2.

The questions were of 2 types: single or multiple-choice questions, and questions assessing the respondent’s agreement with proposals written in the affirmative form, rated on a scale from 0 (not agree at all) to 10 (totally agree). Questions with a score were grouped by domains of the theoretical frameworks (Multimedia Appendix 2).

The questionnaires were tested by 3 patients who were not eligible for the study but had benefited from remote monitoring, and by 3 professionals, focusing on form and content. The estimated time to complete the questionnaire was 15 minutes for both patients and professionals. At the end of the questionnaire, participants were invited to take part in semistructured interviews that explored their use of apTeleCare in detail, based on the 3 categories of factors developed by Jacob et al [17]. We conducted 13 interviews with patients and 11 interviews with health professionals.

### Outcome: Telemonitoring Uptake/Adoption

Among professionals, telemonitoring adoption was defined by the responses to the question, “How often do you consult the telemonitoring application (apTeleCare)?” with 4 response options: “once or several times a day,” “once or several times a week,” “once or several times a month,” and “less often.” The initial recommendation in the NeLLY trial was to use telemonitoring several times a week. Following these recommendations, professionals who reported using the application once a day or once a week were categorized as “frequent users,” while those who used it at least once a month or less often were classified as “average users.” Finally, those who reported not using the telemonitoring application were categorized as “nonusers.” For patients, the type of user (frequent, average, or on-off) was determined after analyzing their application usage data, which was directly extracted from apTeleCare. The initial recommendation in the NeLLY trial was to use telemonitoring at least once a week. Based on this, we considered “frequent users” to be patients with a frequency of use (the number of log-ins divided by application usage time, with usage time defined as the data extraction date minus the training date) greater than or equal to 4 connections per month. Patients with a frequency of use strictly less than 1 connection per month were categorized as “on-off” users. The remaining patients were classified as “average” users.

### Data Collection

An information letter about the study was sent along with the questionnaire to both patients and professionals. Only participants who indicated that they did not object to the research (question 1) were considered.

Patient questionnaire data were initially collected via apTeleCare from March 22 to April 6, 2021. Follow-up emails were sent to patients who did not respond to the application between April 9 and April 26, 2021. Patients had until May 14, 2021, to return the questionnaire. To maintain patient anonymity, questionnaires were first sent to the investigative centers. The clinical research officers at these centers were responsible for entering the patient’s anonymization number on the questionnaire before sending it. Data collected on apTeleCare were securely transmitted to the data controller via TMM Software, the developer of apTeleCare, using a secure file transfer.

Professional questionnaire data were collected using the REDCap database (Research Electronic Data Capture, version 11.0.1; Vanderbilt University) from March 22 to April 17, 2021 [30]. Recruitment was conducted via email, with a link providing access to an online questionnaire hosted by the REDCap database.

Patient and health professional interviews were conducted and transcribed by the first author (MD) between June and August 2021.

The interviews were conducted via videoconference or phone call, based on the participant’s preference, and were audio recorded for accuracy. Nonobjection was obtained orally at the start of each interview.

### Data Analysis

Questionnaire analysis was conducted using R software (R Foundation) [31]. For both populations (patients and professionals), we followed the same analysis plan. First, we described respondent characteristics and the scores obtained for each item. We calculated the number and frequency for categorical variables, and for quantitative variables, we calculated the mean and SD or median, along with the first and third quartiles, depending on the distribution. We also calculated average scores for each domain of our theoretical framework (CFIR and TAM) by summing the scores for the items and dividing by the total number of items in the domain. Once this calculation was performed for all respondents, we calculated the median and first/third quartiles for each domain. Subsequently, we analyzed the consistency of the items by calculating Cronbach α, both globally and by grouping items according to the domains of the theoretical frameworks. We then studied factors associated with telemonitoring adoption using bivariate comparisons with the Wilcoxon Mann-Whitney test among health professionals, grouping them into 2 categories: frequent users and average/on-off users. For patients, we used the Kruskal-Wallis test, grouping them into 3 categories (frequent users, average users, and on-off users). In the professional sample, we analyzed responses according to occupation: nurses or nephrologists, as they were differently involved in telemonitoring. Nurses were responsible for responding to patient alerts, while nephrologists were in charge of including patients and proposing telemonitoring to them.

Interview analysis was conducted by the first and third authors (MD and LT) following a theoretical thematic analysis method [32]. The analysis involved identifying the themes and subthemes raised in the interviews, and then reclassifying them into the different domains of our theoretical frameworks (CFIR and TAM). The analysis was initially conducted separately for interviews with professionals and patients, after which the results were cross-checked between the 2 groups to compare opinions. Verbatims were coded as Nu for nurses, MD for nephrologists, and Pa for patients, with the interview number added (eg, Nu2 corresponds to the second nurse interviewed). These analyses were conducted using NVivo software (Lumivero) [33]. The qualitative analysis report was written by the third author (LT). Key findings from different sources (quantitative questionnaires and semistructured interviews) and different populations (patients and health professionals) were triangulated to understand behavioral determinants and identify barriers and facilitators regarding telemonitoring use in CKD [34].

## Results

### Insights From Health Professionals

#### Sample Characteristics

A total of 43 professionals out of the 103 contacted responded to the questionnaire, representing a response rate of 41.7%. One respondent was excluded because they did not specify whether they were a nurse or a nephrologist. The respondents (nurses and nephrologists) practiced in all 10 centers targeted by the study. Among the 42 respondents, 13 (31%) were nurses and 29 (69%) were nephrologists (Table 1). As shown in Table 1, 8 out of 13 (62%) nurses were classified as “frequent users,” compared with only 1 out of 29 (3%) nephrologists.

**Table 1:** Demographics characteristics of professionals (N=42).

Variables		Total population (N=42)	Nurses (n=13)	Nephrologists (n=29)
			
Number of respondents		42 (100)	13 (31)	29 (69)
**Gender, n (%)**				
	Male	15 (36)	1 (8)	14 (48)
	Female	27 (64)	12 (92)	15 (52)
**Age (years), n (%)**				
	[30; 40[	16 (38)	4 (31)	12 (41)
	[40; 50[	15 (36)	5 (38)	10 (34)
	>50	11 (26)	4 (31)	7 (24)
**Exercise time in the center (months), n (%)**				
	<1	0 (0)	0 (0)	0 (0)
	[2; 6[	1 (2)	1 (8)	0 (0)
	[6; 12[	0 (0)	0 (0)	0 (0)
	>12	41 (98)	12 (92)	29 (100)
**Number of patients included in the NeLLY^a^ trial, n (%)**				
	0	6 (14)	2 (15)	4 (14)
	[1; 5[	15 (36)	3 (23)	12 (41)
	[5; 20[	11 (26)	2 (15)	9 (31)
	>20	10 (24)	6 (46)	4 (14)
**Time of use of the NeLLY service (months), n (%)**				
	<1	1 (2)	1 (8)	0 (0)
	[1; 3[	2 (5)	1 (8)	1 (3)
	>3	18 (43)	11 (85)	7 (24)
	Nonuser	21 (50)	0 (0)	21 (72)
	Total of user	21 (50)	13 (100)	8 (28)
**Use of remote monitoring other than NeLLY, n (%)**				
	Yes, the service uses it	11 (26)	1 (8)	10 (34)
	Yes, I use it	9 (21)	6 (46)	3 (10)
	No	22 (52)	6 (46)	16 (55)
**Frequency of consultation of the application (if used), n/N (%)**				
	At least once a day	4/21 (19)	4/13 (31)	0/8 (0)
	At least once a week	5/21 (24)	4/13 (31)	1/8 (13)
	At least once a month	2/21 (10)	0/13 (0)	2/8 (25)
	Less often	10/21 (48)	5/13 (38)	5/8 (63)
**Type of user, n (%)**				
	Frequent user	9 (21)	8 (62)	1 (3)
	Average user	12 (29)	5 (38)	7 (24)
	One-off user	21 (50)	0 (0)	21 (72)

^a^NeLLY: New Health e-Link in the Lyon Region.

Characteristics by user profile are described in Table 2. The mean age was approximately 44 years in both user groups. Some differences between the 2 user profiles regarding gender and the number of patients included in the NeLLY trial could be linked to the distribution between nurses and nephrologists in the 2 groups. It is noteworthy that 8 of 9 (89%) good users were nurses. Of the 43 respondents, 11 agreed to participate in a semistructured interview, including 4 nephrologists and 7 nurses.

**Table 2:** Description of sociodemographic characteristics according to the user profile in health professionals.

Variables		Frequent users (n=9)	Average/one-off users (n=33)
**Gender, n (%)**			
	Male	1 (11)	14 (42)
	Female	8 (89)	19 (58)
Age (years), mean (SD)		44 (15.95)	44.2 (8.87)
**Type of professional, n (%)**			
	Nephrologists	1 (11)	28 (85)
	Nurses	8 (89)	5 (15)
**Number of patients included in the NeLLY^a^ trial, n (%)**			
	0	0 (0)	6 (18)
	[1; 5[	1 (11)	14 (42)
	[5; 20[	3 (33)	8 (24)
	>20	5 (56)	5 (15)
**Time of use of the NeLLY service (in months)** **, n (%)**			
	<1	0 (0)	1 (3)
	[1; 3[	1 (11)	1 (3)
	>3	8 (89)	10 (30)
	Nonuser	0 (0)	21 (64)

^a^NeLLY: New Health e-Link in the Lyon Region.

#### Consistency of the Questionnaire

The overall Cronbach α coefficient for the professionals’ questionnaire was 0.97, indicating good consistency among all items. All questions related to the TAM showed strong consistency, with coefficients greater than 0.90. Regarding CFIR, 3 domains exhibited weaker consistency: intervention characteristics (Cronbach α=0.55), individual (Cronbach α=0.62), and process (Cronbach α=0.67).

#### Results According to Framework Dimensions

The distribution of the median scores by subdomain of the theoretical framework, profession, and user profile can be found in Tables 3 and 4. The highest scores were observed among respondents in the “Data Security” trust and “Self-Efficacy” perception subdomains of the CFIR (individual field), with respective medians of 8 (IQR 8-10) and 7 (IQR 6-8; Table 3). Indeed, during the interviews, it emerged that health professionals reported using technologies on a daily basis. Nu5 noted the following about using technologies: “In general I manage quite well [...]in fact we use (Information technologies) all the time for patient medical records, telemonitoring, e-mail communication”. The least-rated subdomains were Perceived Adaptability, Perceived Response to Patients’ Needs, Peer Support, and Planning. Adaptability, referring to the integration of telemonitoring into daily workflow, was perceived by the majority of nurses as an additional workload (Nu2: “It takes work you, don’t realize how long it takes to manage an alert”). However, in a center where they dedicated a nurse to the telemonitoring activity, the experience was different (Nu7: “Frankly, no, I don’t think so (increase in workload). You have to contact those who don’t log on, but I think that’s part of the role of the professional behind the application. I don’t see that as a constraint, but as my role”). Peer support for using the application was perceived as insufficient, as mentioned by Nu4 “I asked for support from my colleagues who had also been trained in NeLLY, so that I could take over, except that it fell apart over time, [...] At first my colleagues took over (checking alerts when Nu4 was off) and lately there’s no longer any relay.” Regarding the Planning subdomain (organizational processes and protocols to integrate the application into practice), interviewees expressed regret over the lack of a structured protocol to support them in providing information and training to patients on how to use the telemonitoring system.

**Table 3:** Description of scores according to the type of professional (nurse or nephrologist) and the field of theoretical frameworks (N=42).

Domain and subdomain (CFIR^a^)			Total population (N=42), median (Q1-Q3)		Nurses (n=13), median (Q1-Q3)		Nephrologists (n=29), median (Q1-Q3)		*P* value^b^
**Intervention characteristics**									
	Adaptability	5 (4-7)		5 (4-6)		6 (3-7)		.94	
**Outer settings**									
	Patients’ Needs and Resources	5 (4-6)		5 (4-5)		5 (4-6)		.35	
	Peer Pressure	5 (3-6)		4 (3-6)		5 (3-6)		.54	
**Inner settings**									
	Readiness for Implementation	5 (4-6)		5 (3-7)		5 (4-6)		.78	
**Individual**									
	Data Security	8 (8-10)		8 (8-10)		9 (8-10)		.97	
	Self-Efficacy	7 (6-8)		7 (7-8)		7 (6-8)		.49	
**Process**									
	Planning	5 (3-9)		4 (3-5)		8 (6-10)		<.01	
**TAM^c^**									
	Perceived Ease of Use	6 (5-8)		6 (5-7)		6 (5-8)		.93	
	Perceived Usefulness	7 (5-7)		7 (6-8)		7 (5-7)		.35	
	Behavioral Intention to Use	6 (3-8)		6 (4-8)		7 (2-8)		.60	

^a^CFIR: Consolidated Framework for Implementation Research.

^b^*P* value for the comparison between the 2 users’ profiles (Wilcoxon test).

^c^TAM: Technology Acceptance Model.

**Table 4:** Descriptions of scores based on user profile and areas of theoretical frameworks in health professionals (N=42).

Field and subdomain (CFIR^a^)			Frequent user (n=9), median (Q1-Q3)	Average/on-off user (n=33), median (Q1-Q3)	*P* value^b^
**Characteristics of the intervention**					
		Adaptability	6 (5-8)	4 (3-6)	.02
**Outer settings**					
		Patients’ Needs and Resources	5 (5-6)	5 (3-6)	.92
		Peer Pressure	6 (4-7)	5 (3-6)	.18
**Inner settings**					
		Readiness for Implementation	7 (5-8)	5 (4-6)	.07
**Individual**					
		Data Security	9 (8-10)	8 (7-10)	.45
		Self-Efficacy	8 (7-9)	7 (5-8)	.05
**Process**					
		Planning	5 (4-9)	5 (3-8)	.89
**Field (TAM^c^)**					
	Perceived Ease of Use		7 (6-8)	6 (5-8)	.38
	Perceived Usefulness		8 (7-8)	6 (5-7)	.008
	Behavioral Intention to Use		8 (7-9)	5 (2-7)	.02

^a^CFIR: Consolidated Framework for Implementation Research.

^b^*P* value for the comparison between the 2 users’ profile (Wilcoxon test).

^c^TAM: Technology Acceptance Model.

#### Results According to Profession and User Profile

Looking at the scores by profession (Table 3), nurses generally had equal or lower ratings than nephrologists. Two domains were rated below 5/10 by nurses: Peer Support and Planning. The Planning subdomain showed a significantly lower score for nurses compared with nephrologists, with a median of 4 (IQR 3-5) versus 8 (IQR 6-10), respectively (*P*<.01). Concerns in the Planning subdomain were explained in interviews regarding the collaboration between nurses and nephrologists in managing telemonitoring alerts. Nu1 mentioned, “I monitor, I alert but if I don’t get feedback from the medical team I can’t do much more.” Peer support was less well perceived among nurses (Table 3) who considered that telemonitoring was not a priority for the medical team, Nu2 “We sent an alert to a doctor, by e-mail for example, and got no response. [...] It (telemonitoring) is not yet recognized as a priority.”, and that only few professionals really got involved, Nu3 “In a 10-person medical team, only one person is really involved.”

Table 4 shows the median scores among health care professionals by domain according to the user profile. The details of the scores by question are available in Multimedia Appendix 3. Significant differences according to user profiles were observed in the Adaptability subdomain of CFIR (median 6, IQR 5-8 vs median 4, IQR 3-6, *P*=.02), Perceived Usefulness (median 8, IQR 7-8 vs median 6, IQR 5-8, *P*=.008), and Behavioral Intention to Use (median 8, IQR 7-9 vs median 5, 2-7, *P*=.02) of the TAM, with median scores 2-3 points lower for average users compared with frequent users. Questions in the Adaptability subdomain of CFIR focused on workload and changing practices. In the interviews, professionals linked the workload to the management of apTeleCare. Nu4 mentioned, when discussing technical issues, “It worked well at first, but then the problems started with a large number of patient calls*.*” The perceived usefulness of telemonitoring for patients was mentioned during the interviews. Nu7 said, “It’s really something that can be very beneficial for the patient, allowing us to have contact with them, a very close follow-up [...] in the kidney disease, these are the patients with whom we need to stay in regular contact to be able to avoid any problems.”

To a lesser extent, average users tended to have lower scores for the Readiness for Implementation (median 7, IQR 5-8 vs median 5, IQR 4-6, *P*=.07) and Self-Efficacy (median 8, IQR 7-9 vs median 7, IQR 5-8, *P*=.05) domains of the CFIR. The readiness for implementation questions focused on the management of the application from a technical point of view.

### Insights From Patients

#### Sample Characteristics

A total of 128 patients out of 305 contacted responded to the questionnaire, representing a response rate of 42.0%. No statistically significant differences were found between the demographic characteristics of patients who responded to the survey and nonrespondents from the source population (Multimedia Appendix 4). Respondents were from all 10 centers in the intervention period, which had already included patients. Of these respondents, 75 (58.6%) completed the survey via apTeleCare and 53 (41.4%) via postal mail. The mean age of the population was 70.8 (SD 10.2) years. As shown in Table 5, 46 (35.9%) patients were classified as “frequent users” (≥4 connections/month), 39 (30.5%) as “average users,” and 15 (11.7%) as “on-off users” (<1 connection/month). The mean age of frequent users was approximately 74.3 years, which is similar to that of the other 2 groups. Some differences were observed regarding gender, center, and the presence of a caregiver across the profiles.

**Table 5:** Demographic characteristics of patients in total population and by type of user (N=128).

Variables		Total population (N=128)	Frequent user (n=46)	Average user (n=39)		One-off user (n=15)
**Questionnaire format, n (%)**						
	apTeleCare	75 (58.6)	41 (89.1)	26 (66.7)	1 (6.7)	
	Mail	53 (41.4)	5 (10.9)	13 (33.3)	14 (93.3)	
**Gender, n (%)**						
	Male	85 (66.4)	33 (71.7)	22 (56.4)	10 (66.7)	
	Female	43 (33.6)	13 (28.3)	17 (43.6)	5 (33.3)	
**Age (years)**						
	Mean (SD)	71.9 (10.2)	74.3 (9.87)	68.85 (10.9)	71.5 (11.1)	
	Range	39-94	47-94	39-86	50-87	
**Monitoring center, n (%)**						
	Centre Hospitalier Universitaire (University hospitals centers)	61 (47.7)	21 (45.7)	16 (41.0)	9 (60.0)	
	Nonuniversity hospitals/association center	67 (52.3)	25 (54.3)	23 (59.0)	6 (40.0)	
**NeLLY^a^ service proposal date (months), n (%)**						
	<1	4 (3.1)	1 (2.2)	0 (0)	1 (6.7)	
	[1; 3[	7 (5.5)	4 (8.7)	0 (0)	0 (0)	
	[3; 6[	20 (15.6)	5 (10.9)	6 (15.4)	4 (26.7)	
	≥6	86 (67.2)	36 (78.3)	33 (84.6)	9 (60.0)	
**Frequency of use of a computer/tablet/smartphone, n (%)**						
	Daily	101 (78.9)	38 (82.6)	30 (76.9)	13 (86.7)	
	Weekly	12 (9.4)	3 (6.5)	5 (12.8)	1 (6.7)	
	Less often	10 (7.8)	4 (8.7)	2 (5.1)	0 (0)	
	Never	5 (3.9)	1 (2.2)	2 (5.1)	1 (6.7)	
**Frequency of e-mailbox consultation, n (%)**						
	Daily	98 (76.6)	35 (76.1)	29 (74.4)	11 (73.3)	
	Weekly	17 (13.3)	4 (8.7)	6 (15.4)	4 (26.7)	
	Less often	9 (7.0)	6 (13.0)	2 (5.1)	0 (0.0)	
	Never	4 (3.1)	1 (2.2)	2 (5.1)	0 (0.0)	
**Internet connection speed, n (%)**						
	Very good	50 (39.1)	20 (43.5)	13 (33.3)	7 (46.7)	
	Pretty good	41 (32.0)	16 (34.8)	13 (33.3)	3 (20.0)	
	Medium	25 (19.5)	8 (17.4)	9 (23.1)	3 (20.0)	
	Bad	10 (7.8)	2 (4.3)	3 (7.7)	2 (13.3)	
**Help of caregiver, n (%)**						
	Yes	32 (25.0)	11 (23.9)	15 (38.5)	3 (20.0)	
	No	67 (52.3)	32 (69.6)	21 (53.8)	6 (40.0)	
**If yes, what kind of caregivers?^b^, n (%)**						
	Family members or relatives	16 (50.0)	7 (63.6)	8 (53.3)	1 (33.3)	
	Caregiver of nephrology service	16 (50.0)	4 (36.4)	7 (46.7)	2 (66.7)	
**Remote monitoring improves, n (%)**						
	Relationships with health professionals^c^	96 (75.0)	41 (89.1)	30 (76.9)	8 (53.3)	
	Quality of medical monitoring	58 (45.3)	21 (45.7)	25 (64.1)	6 (40.0)	
	The course of kidney disease	57 (44.5)	28 (60.9)	15 (38.5)	4 (26.7)	
	The attention I pay to my state of health	53 (41.4)	24 (52.2)	16 (41.0)	4 (26.7)	
	Quality of care	34 (26.6)	15 (32.6)	10 (25.6)	4 (26.7)	
	Considering the experience of my illness	31 (24.2)	7 (15.2)	12 (30.8)	3 (20.0)	
	Taking my medication	17 (13.3)	6 (13.0)	6 (15.4)	1 (6.7)	
**Reasons which help to continue the use of remote monitoring, n (%)**						
	I see an interest in my health	61 (47.7)	1 (2.2)	1 (2.6)	4 (26.7)	
	A health professional at my center recommended it to me	52 (40.6)	22 (47.8)	24 (61.5)	7 (46.7)	
	It saves time	5 (3.9)	4 (8.7)	13 (33.3)	6 (40.0)	
	My relatives recommended it to me	8 (6.3)	4 (8.7)	1 (2.6)	0 (0)	
	I do not use remote monitoring	4 (3.1)	5 (10.9)	3 (7.7)	0 (0)	

^a^NeLLY: New Health e-Link in the Lyon Region.

^b^Proportion calculated on patients who said yes in different groups.

^c^Proportion calculated on patients who said yes to either improve communication with a nephrologist or a nurse or relationship with health professionals in general.

Regarding patients’ perceptions of telemonitoring, 96 (75%) patients felt that it improved their relationship with health professionals. Additionally, across the total population and within each user group, patients agreed that telemonitoring enhanced their attention to their disease. Furthermore, approximately half of the patients believed that telemonitoring improved the quality of their medical monitoring (58/128, 45.3%) and the course of their kidney disease (57/128, 44.5%). The main reasons for nonuse, as reported by on-off users, were a lack of interest in telemonitoring (8/25, 32%), difficulties in using the telemonitoring application (7/25, 28%), and internet connection issues (6/25, 24%). Otherwise, in the total patient population, 32 (25%) reported using telemonitoring with the help of a caregiver. No statistically significant differences were identified between users regarding the variables associated with the use of remote monitoring (Multimedia Appendix 5). Further general demographics can be found in Table 5.

Of the 128 respondents, 13 accepted to participate in a semistructured interview (8 men). The mean age of the participants was 67.5 years. Regarding the user profiles, 5 were frequent users, 2 were average users, 1 was an on-off user, and 4 were not categorized because their usage data were unavailable.

#### Consistency of the Questionnaire

The overall Cronbach α coefficient for the patient questionnaire was 0.91, indicating good consistency between all items. Questions relating to TAM showed good consistency with coefficients around 0.90. Similar to the professionals’ questionnaire, the domains with less strong consistency were from the CFIR framework: Individual (Cronbach α=0.25) and Outer Settings (Cronbach α=0.59).

#### Results According to Framework Dimensions

The median scores by domains and subdomains of our 2 theoretical frameworks in the total population were generally high (median ≥7). Higher scores were observed for the Data Security (knowledge and beliefs; median 10) and Peer Support (median 8) domains (Table 6). During the interviews, every patient mentioned receiving support in the use of telemonitoring from their relatives, although not necessarily related to their disease. Indeed, some patients do not discuss their disease out of fear of bothering their loved ones. As Pa2 mentioned, “No, if I have an internet problem I call one of my daughters, but no, no I never talk about my health. You can’t leave with your illness all the time.” Similarly, Pa11 said, “I try not to spread my illness and then my entourage is bored enough.” Regarding the knowledge and belief aspect, patients were generally unconcerned about the use of their data, as they understood it was being used by their health care professionals. As Pa2 mentioned, “because it helps Professor Y (nephrologist) every time he sees me.”

**Table 6:** Descriptions of scores based on user profile and areas of theoretical frameworks in patients (N=100).

Domain and subdomain (CFIR^a^)			Total population (N=128), median (Q1-Q3)		Frequent user (n=46), median (Q1-Q3)		Average user (n=39), median (Q1-Q3)		One-off user (n=15), median (Q1-Q3)		*P* value^b^		
**Outer Settings**													
	Peer Pressure	8 (8-9)		7 (5-9)		8 (4-10)		8 (8-9)		.55			
**Inner Settings**													
	Readiness for Implementation	8 (7-9)		8 (5-8)		8 (5-10)		8 (6-9)		.97			
**Individual**													
	Data Security	10 (9-10)		9 (5-10)		10 (9-10)		8 (0-10)		.09			
	Self-Efficacy	8 (6-8)		7 (5-10)		8 (5-9)		8 (1-8)		.51			
**TAM^c^**													
	Perceived Ease of Use	8 (5-9)		9 (8-10)		9 (7-10)		5 (4-9)		.44			
	Perceived Usefulness	7 (5-9)		7 (6-9)		8 (5-9)		6 (5-7)		.87			
	Behavioral Intention to Use	8 (3-9)		7 (5-10)		7 (5-10)		7 (0-9)		.38			

^a^CFIR: Consolidated Framework of Implementation Research.

^b^*P* value for the comparison between the three users’ profile (Kruskal-Wallis test).

^c^TAM: Technology Acceptance Model.

#### Results According to the User Profile

Looking at the scores by user profile (Table 6), median scores across all user groups were close and high. Although the differences were not statistically significant, some variations were observed between the user profiles (Table 6). On-off users, in particular, had wider score distributions compared with the other groups, with lower first quartile values in the Data Security and Self-Efficacy subdomains of the CFIR, as well as in the Perceived Ease of Use and Behavioral Intention to Use domains of the TAM. Questions regarding self-efficacy focused on the ease of use of the internet. In the interviews, 6 out of 13 patients mentioned feeling uncomfortable with computers. Pa13 shared his experience with using a new computer: “It may have been simpler for the others, but for me it was complicated, I had trouble, I still have trouble using it so in the end I'm going to use it less and less.” The perceived ease of use of telemonitoring was a common theme in the interviews; 9 out of 13 patients reported having difficulty using apTeleCare, and 10 out of 13 encountered technical problems. However, only 3 out of 13 had their technical issues resolved. Technical issues mentioned during the interviews included bugs and problems with log-ins and passwords. Pa4 explained, “at one point when I tried to set the measurements such as blood pressure, each time it did not work so I had to disconnect, then reconnect again.” Pa11 shared, “there was an update that caused a system bug and as I had contacted the local correspondent [...], she sent them to the hotline who had to call me back to solve the problem and then I never got a reply.”

### Insights Crossed Between Professionals and Patients

Several topics were discussed by both health care professionals and patients during the interviews. Both groups highlighted the perceived psychological benefits of telemonitoring for patients and the overall improvement in patient care. Regarding the psychological benefits, professionals reported that patients often expressed feeling reassured through their interactions. As MD3 mentioned, “Feedbacks from patients is that they feel reassured.” Nu2 also shared, “Yeah, like the words some people say (Nu2cites patients feedback that she collected during telemonitoring calls): ‘it’s comforting’, ‘it’s a positive approach’, ‘it’s a weekly constraint that encourages us to listen better, detect the warning signs of a possible evolution of our pathology’.” This sense of safety expressed by professionals was also echoed by patients, who reported a reduction in anxiety related to their disease: “It’s already more reassuring, since you know that you have someone immediately in case of a problem.” [Pa6]. The evolution of patient care was also highlighted, with several mentions of the responsiveness in case of issues. As Pa8 said, “in case of difference, the doctor can intervene.” Another key point raised by both professionals and patients was patient empowerment. The adoption of a greater awareness of their disease was noted, with Pa5 stating, “it forces you to be vigilant, it forces you to understand a little about your disease too, [...] to know what you can do and what you cannot do. [...] we feel responsible for ourselves.” However, the perceived ease of use of telemonitoring was impacted by technical issues such as “bugs,” reconnection problems, and password issues, which were mentioned frequently during the interviews. As Pa1 noted, “I don’t find it very ergonomic. [...] It is not easy to use, you can very easily end up bothering with passwords. [...] There are two portals one behind the other [...] in reality the second portal disappears automatically but it disappears after 30 seconds. Then it’s over.” Finally, one factor mentioned by health care professionals that may have impacted patients was the adaptation of the application’s usage frequency in their centers. As Nu2 explained, “We negotiated with them, we tried to find out why they weren’t logging on every week, they told us it was burdensome; so, we suggested they lighten up, fill in once every 15 days.”

## Discussion

### Principal Findings

This ancillary study aimed to identify the barriers and facilitators in implementing a telemonitoring application for patients with CKD and health professionals. Although telemonitoring adoption was generally positive, through interviews with our populations and using both quantitative and qualitative approaches, we identified several areas for improvement.

Professionals were divided into 2 groups with distinct roles: nephrologists prescribed the telemonitoring, while nurses were responsible for following up with telemonitoring patients. This confirms that telemonitoring use depends on the occupation—nephrologist or nurse [20]. Indeed, the majority of nephrologists (28/29) were average or nonusers of telemonitoring, whereas most nurses (8/13) were frequent users. Some authors [20,21] distinguished the roles that nurses and physicians should play in the implementation of telemonitoring. Nurses were considered more adaptable to this new “working method” and were seen as key players due to their close relationship with patients. Our results reveal several factors that can be interpreted as facilitators for improving the use of telemonitoring among professionals. These facilitators include confidence in the security of the data collected by the application, its ease of use, and its perceived usefulness for patients. Differences were also observed across professional roles and user types in other domains. The scores in the Adaptability subdomain of CFIR, which related to workload and the time required to integrate telemonitoring into existing practice, were lower in the average/nonuser group. This issue was also mentioned repeatedly during the interviews and appears to particularly impact nurses’ workloads. Modifying workload is a key factor in the adoption of telemonitoring. Indeed, both the time required for use and the ease of use can significantly affect workload, either positively (by decreasing it) or negatively (by increasing it) [17,20,21]. This issue can be linked to other subdomains of CFIR, such as readiness for implementation and planning. The results indicated that nurses felt there was no well-defined nursing protocol in their center for the use of telemonitoring. Several studies highlight the importance of having proper training and dedicated spaces for telemonitoring [20,22]. Implementing a training and support protocol for professionals, in alignment with their workload and availability, is a crucial point to consider if we aim to improve adherence to telemonitoring among health professionals [20,21,28].

The patient population in our study had a high average age, which may explain some difficulties in using the telemonitoring application [16,21]. However, there was no significant age difference between the various user profiles in our study. This effect warrants further investigation in a larger population or with a wider age range. The instructions for using the NeLLY service were to complete 1 questionnaire per week, but based on the qualitative interviews, health professionals adapted the frequency based on the needs of their patients in their centers. A total of 46 (35.9%) patients completed their questionnaires at least once a week as recommended for the telemonitoring program. Several barriers and facilitators were identified in our results, which align with existing literature. Our findings indicated that the ease of use of the telemonitoring system was perceived as high, which could serve as a facilitator for its use [22,35]. Additionally, the perceived usefulness of telemonitoring, particularly if patients believe it can improve their health or help them gain more independence in managing their disease, appears to have a positive impact on its adoption [12]. Moreover, almost half of the patients across all user groups felt that telemonitoring helped them pay more attention to their condition. Several studies have shown that telemonitoring can enhance patients’ self-management skills and empower them [23,35,36]. Peer support also emerged as a potential lever for encouraging the use of the telemonitoring application. All patients interviewed during the qualitative phase mentioned receiving technical assistance or support from their relatives. Additionally, the support from professionals and family members was highlighted in the questions. Consistent with this, the literature emphasizes that the engagement of health care professionals with their patients plays a crucial role in the successful implementation of telemonitoring programs [22,37]. Finally, our results showed that some on-off users were less comfortable with the computer tool (self-efficacy) and lacked motivation to use it (behavioral intention to use). These patients also had lower scores for the ease of use of the telemonitoring system; bugs, connection issues, and password problems were frequently mentioned during the interviews. Such technical issues could have further diminished their intention to continue using telemonitoring. Technical problems were also identified among professionals, impacting their workload. This aspect has been previously recognized in the literature as a barrier, as it affects the ease of use of the device [17]. However, the results are exploratory, and the questionnaires developed did not capture the personal context of the participants, which could influence self-efficacy and, consequently, the behavior of the participants [38]. Therefore, in this context, it is valuable to combine mixed methods and 2 theoretical frameworks (CFIR and TAM) to obtain a more comprehensive understanding and explore the real-life context of the participants through qualitative interviews.

The results suggest the need for tailored implementation strategies that address the specific issues faced by both patients and professionals, as they share common and distinct challenges. However, professional acceptance is crucial, as health care professionals’ perceptions and the support they offer directly influence patients’ adoption of remote monitoring. Training should also emphasize the patient-professional relationship and the professional’s approach when prescribing telemonitoring. The role of a dedicated facilitator, as proposed in the integrated-Promoting Action on Research Implementation in Health Services (iPARIHS) implementation framework [39], could be beneficial in offering continuous reinforcement and assisting professionals in integrating the innovation into their daily practice. This approach could include strategies tailored to both professionals and patients.

### Limitations

This study has 2 key limitations. First, due to the ongoing COVID-19 health crisis, we were unable to validate our questionnaires through focus groups. Bringing together patients with CKD during this time posed an unnecessary risk to their health. To address this challenge, we tested the patient questionnaire individually with 3 patients and 3 professionals, which allowed us to make necessary adjustments to both the form and content. Our second limitation was the small sample size of professionals. While the response rate was relatively high compared with the literature, it was too low to allow for meaningful comparisons or to provide sufficient statistical power for multivariate regression models. As a result, we were unable to quantify the strength of the associations between the identified barriers and facilitators and the use of telemonitoring. These factors were explored during semistructured interviews with nephrologists and nurses. Additionally, the survey predominantly reached frequent and average users, with only 15 participants identified as irregular users. Therefore, we can assume that the barriers identified in our sample are likely to be more significant for the broader population of irregular users.

### Conclusions

This study highlighted that patients in the NeLLY trials generally adhered to telemonitoring. Among health care professionals, however, adoption varied, with nurses showing higher levels of adherence compared with nephrologists.

Nurses and nephrologists were involved in telemonitoring with patients with CKD in very different ways. Despite these distinct roles, both groups faced common barriers to the implementation of telemonitoring, such as the additional workload generated by remote monitoring. Addressing these challenges is essential to improve the adoption of telemonitoring among health care professionals. Given that support from health care professionals is crucial for patient adoption of telemonitoring, it is important to target both health care professionals and patients to enhance the implementation of telemonitoring in CKD. These factors should be taken into account when interpreting the results of the main cost-effectiveness study (PRME NeLLY) and underscore the need for trials that also evaluate the efficacy of implementation strategies.

## Appendix

Multimedia Appendix 1 apTelecare log-in page.

Multimedia Appendix 2 Details of questions by theoretical framework in patient’s and professional's questionnaire.

Multimedia Appendix 3 Details of scores by questions among health professionals.

Multimedia Appendix 4 Complementary analysis.

Multimedia Appendix 5 Comparison of variables linked to technological literacy between different types of users.

## Abbreviations

CFIR: Consolidated Framework for Implementation Research
CKD: chronic kidney disease
iPARIHS: integrated-Promoting Action on Research Implementation in Health Services
NeLLY: New Health e-Link in the Lyon Region
PRME: Medical and Economic Research Program
REDCap: Research Electronic Data Capture
SW-RCT: stepped-wedge randomized controlled trial
TAM: Technology Acceptance Model
