# Supplementary figures and images for: Barriers and Facilitators in Implementing a Telemonitoring Application for Patients With Chronic Kidney Disease and Health Professionals: Ancillary Implementation Study of the NeLLY (New Health e-Link in the Lyon Region) Stepped-Wedge Randomized Controlled Trial
Source: JMIR Mhealth Uhealth. 2025 Jan 22;13:e50014. doi: 10.2196/50014 (PMC11799818; doi:10.2196/50014)

A. ApTelecare login page – patient interface


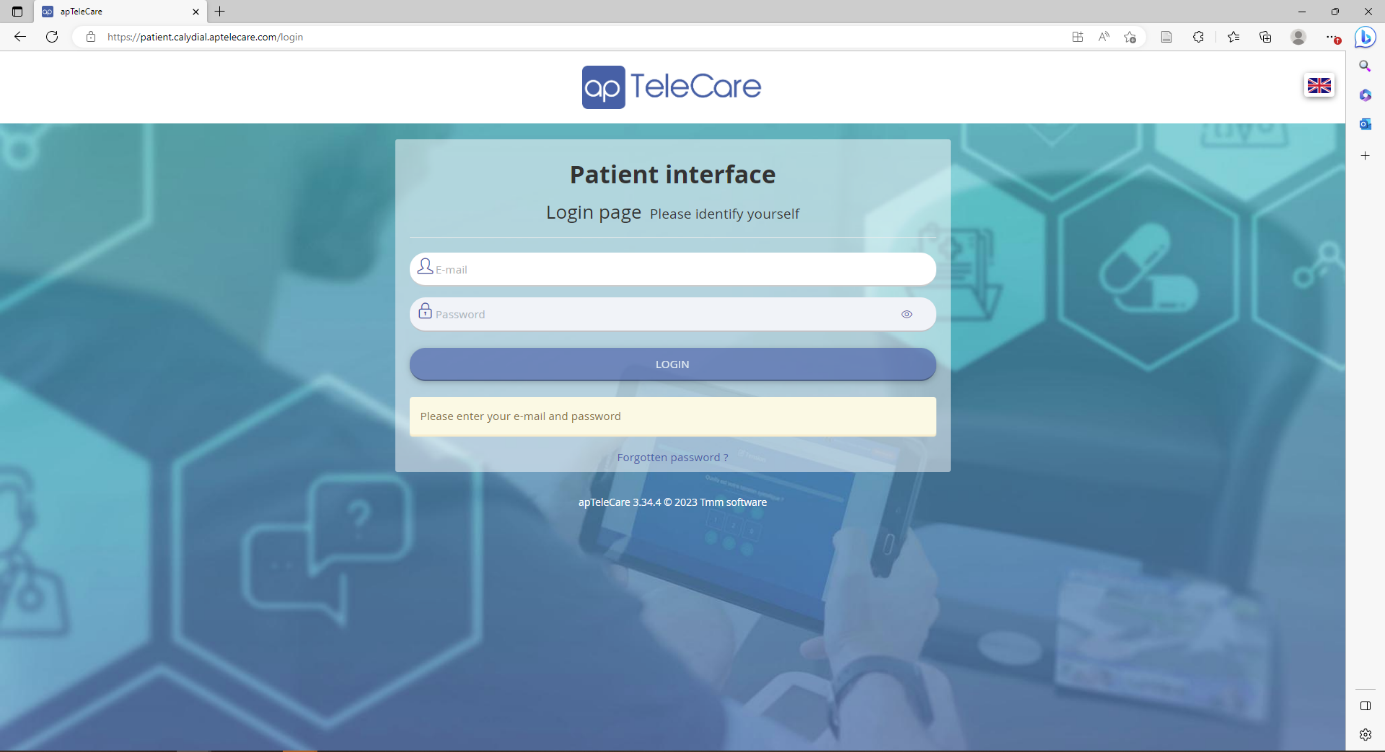


B. ApTelecare login page – professional interface


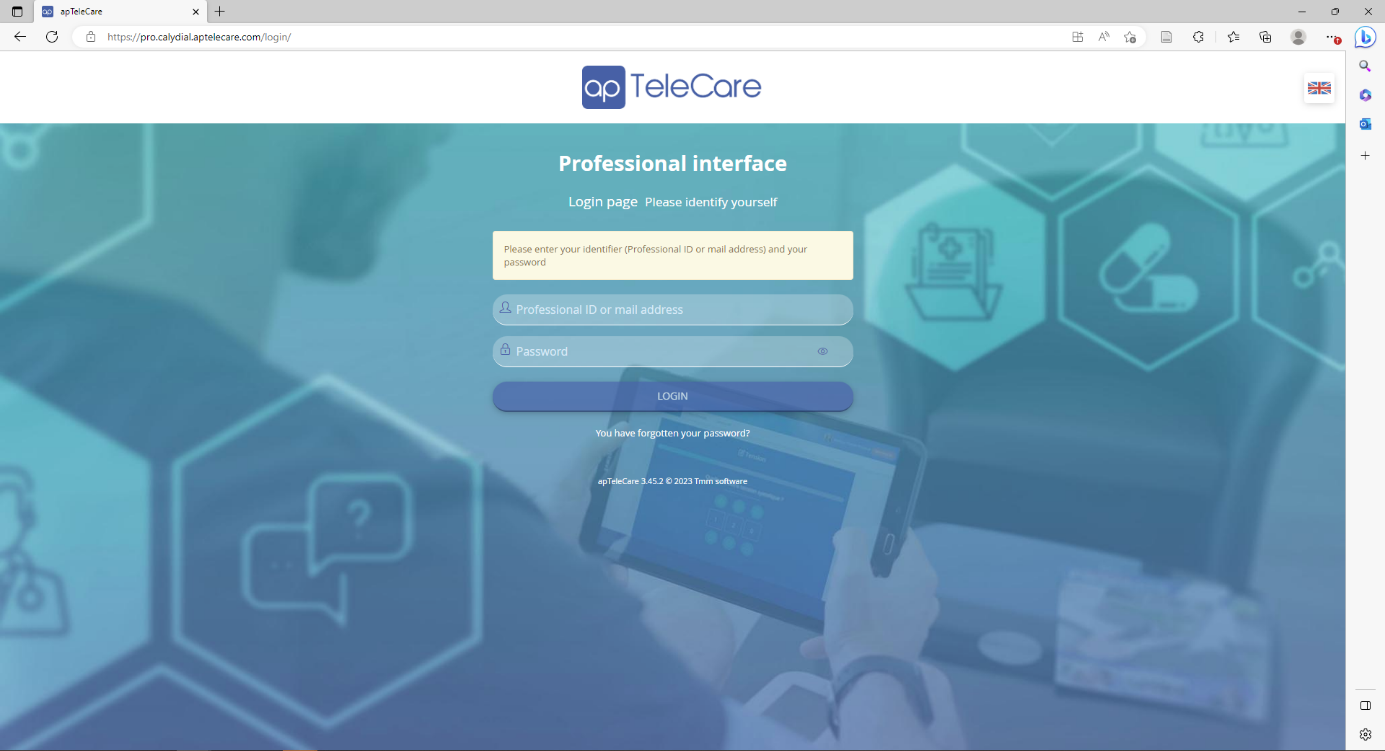

Supplement: Multimedia Appendix 1 [file mhealth_v13i1e50014_app1.docx]
