# Supplementary material for: Barriers and Facilitators in Implementing a Telemonitoring Application for Patients With Chronic Kidney Disease and Health Professionals: Ancillary Implementation Study of the NeLLY (New Health e-Link in the Lyon Region) Stepped-Wedge Randomized Controlled Trial
Source: JMIR Mhealth Uhealth. 2025 Jan 22;13:e50014. doi: 10.2196/50014 (PMC11799818; doi:10.2196/50014)
Supplement: Multimedia Appendix 2 [file mhealth_v13i1e50014_app2.docx]

1. Details of questions by theoretical framework in patient’s questionnaire.

| **Theoretical Framework** | | Question No. | Question Statement |
| --- | --- | --- | --- |
| **Domain (CFIR*)** | **Subdomain (CFIR*)** |  |  |
| Outer settings | Peer pressure | 25 | My family and friends support me in my use of telemonitoring. |
|  |  | 26 | Health professionals support my use of telemonitoring |
| Inner settings | Readiness for implementation | 24 | I have the necessary skills to use telemonitoring. |
| Individual | Knowledge and Belief about the intervention | 13 | The confidentiality and security of my personal data are respected with the telemonitoring application. |
|  | Self-efficacy | 14 | I'm comfortable with computer tools in general |
| **Domain (TAM**)** | | | |
| Perceived Ease of Use | | 5 | NeLLY telemonitoring service is easy for patients to use |
|  |  | 6 | Telemonitoring is compatible with regular use |
|  |  | 9 | The telemonitoring application is easy to use |
| Perceive Usefulness | | 10 | The different functions offered by the telemonitoring application meet my expectations and/or needs for my telemonitoring. |
|  |  | 11 | Overall, I am satisfied with the telemonitoring application. |
|  |  | 12 | Overall, I'm satisfied with my telemonitoring service. |
|  |  | 18 | Telemonitoring is useful for improving my health |
|  |  | 21 | Telemonitoring reassures me about monitoring my kidney disease |
|  |  | 22 | Telemonitoring was used during the COVID-19 health crisis |
|  |  | 23 | Telemonitoring provided reassurance during the COVID-19 health crisis |
| Behavioral Intention to Use | | 25 | I’m motivated to use telemonitoring |
| **CFIR is the Consolidated Framework for Implementation Research; **TAM is the Technology Acceptance Model* | | | |

B. Details of questions by theoretical framework in professional’s questionnaire.

| **Theoretical framework** | | Question No. | Question statement |
| --- | --- | --- | --- |
| **Domain (CFIR*)** | **Subdomain (CFIR*)** |  |  |
| Intervention characteristics | Adaptability | 14 | Telemonitoring has changed my practices. |
|  |  | 31 | My daily workload has been modified by the introduction of the NeLLY service. |
|  |  | 34 | I have the time I need to integrate telemonitoring into my practice (patient training and alert follow-up). |
| Outer settings | Patients’ needs and resources | 37 | Patients are motivated to use telemonitoring. |
|  |  | 38 | Patients with chronic kidney disease are sufficiently comfortable with computer tools to use telemonitoring. |
|  | Peer pressure | 25 | My colleagues support me in implementing telemonitoring. |
|  |  | 27 | The other healthcare professionals at my center use telemonitoring in their daily practices. |
|  |  | 28 | Other healthcare professionals involved in monitoring chronic kidney disease use telemonitoring. |
| Inner settings | Readiness for implementation | 13 | ApTelecare technical support will provide you with the assistance you need if you have any problems with the ApTelecare tool. |
|  |  | 26 | The management of my center or department supports me in implementing remote monitoring. |
|  |  | 30 | In general, healthcare professionals are well trained to use telemonitoring. |
|  |  | 42 | The department in which I work has the resources needed to implement remote monitoring. |
| Individual | Knowledge and belief about the intervention | 12 | The confidentiality and security of patient data collected in the NeLLY service are respected. |
|  | Self-efficacy | 23 | I'm comfortable with computer tools in general. |
|  |  | 24 | I feel comfortable using telemonitoring. |
| Process | Planning | 33 | A nursing protocol is in place for remote monitoring in my center. |
|  |  | 36 | Alerts are handled in collaboration between the nurse and the nephrologist. |
| **Domain (TAM**)** | | | |
| Perceived Ease of Use | | 9 | The telemonitoring application is easy to use for healthcare professionals |
|  |  | 10 | The following functions are easy to use: |
|  |  | 10_a | Connection |
|  |  | 10_b | Setting |
|  |  | 10_c | Alert follow-up and resolution |
|  |  | 10_d | Use of the messaging system |
|  |  | 10_e | Teleconsultation |
|  |  | 11 | Telemonitoring is compatible with my current practice. |
|  |  | 35 | It's easy to interpret and process alerts received via the remote monitoring application. |
| Perceive Usefulness | | 16 | Telemonitoring improves continuity of care. |
|  |  | 15 | Telemonitoring improves communication and patient relations |
|  |  | 17 | Telemonitoring improves the quality of patient care. |
|  |  | 19 | Telemonitoring improves patient health |
|  |  | 20 | Telemonitoring slows down the progression of patients' kidney disease. |
|  |  | 21 | Telemonitoring allows for more prevention. |
|  |  | 22 | Telemonitoring helps empower patients. |
|  |  | 32 | Telemonitoring has changed the way we work as a team. |
| Behavioral Intention to Use | | 39 | By the time telemonitoring was implemented, I was ready to use it. |
|  |  | 40 | Today, I'm ready to use telemonitoring with my patients. |
|  |  | 41 | Today, I would recommend the use of telemonitoring to other healthcare professionals. |
| **CFIR is the Consolidated Framework for Implementation Research; **TAM is the Technology Acceptance Model* | | | |
