# Supplementary material for: Barriers and Facilitators in Implementing a Telemonitoring Application for Patients With Chronic Kidney Disease and Health Professionals: Ancillary Implementation Study of the NeLLY (New Health e-Link in the Lyon Region) Stepped-Wedge Randomized Controlled Trial
Source: JMIR Mhealth Uhealth. 2025 Jan 22;13:e50014. doi: 10.2196/50014 (PMC11799818; doi:10.2196/50014)
Supplement: Multimedia Appendix 3 [file mhealth_v13i1e50014_app3.docx]

Details of scores by questions among health professionals

| **Question No.** | **Question statement** | **N** | **Median** | **Q1** | **Q3** |
| --- | --- | --- | --- | --- | --- |
| **9** | The telemonitoring application is easy to use for healthcare professionals | 22 | 7 | 5 | 8 |
| **10** | The following functions are easy to use: | | | | |
| **10_a** | Connection | 22 | 5 | 2 | 7 |
| **10_b** | Setting | 22 | 7 | 4 | 8 |
| **10_c** | Alert follow-up and resolution | 22 | 6 | 5 | 8 |
| **10_d** | Use of the messaging system | 22 | 8 | 6 | 9 |
| **10_e** | Teleconsultation | 22 | 5 | 2 | 8 |
| **11** | Telemonitoring is compatible with my current practice. | 41 | 7 | 5 | 8 |
| **12** | The confidentiality and security of patient data collected in the NeLLY service are respected. | 22 | 9 | 8 | 10 |
| **13** | ApTelecare technical support will provide you with the assistance you need if you have any problems with the ApTelecare tool. | 22 | 7 | 5 | 8 |
| **14** | Telemonitoring has changed my practices. | 22 | 6 | 4 | 8 |
| **15** | Telemonitoring improves communication and patient relations | 41 | 6 | 5 | 8 |
| **16** | Telemonitoring improves continuity of care. | 41 | 7 | 5 | 9 |
| **17** | Telemonitoring improves the quality of patient care. | 41 | 7 | 5 | 8 |
| **19** | Telemonitoring improves patient health | 41 | 5 | 5 | 7 |
| **20** | Telemonitoring slows down the progression of patients' kidney disease. | 41 | 5 | 5 | 7 |
| **21** | Telemonitoring allows for more prevention. | 41 | 7 | 5 | 8 |
| **22** | Telemonitoring helps empower patients. | 41 | 7 | 6 | 9 |
| **23** | I'm comfortable with computer tools in general. | 41 | 8 | 6 | 9 |
| **24** | I feel comfortable using telemonitoring. | 41 | 6 | 4 | 8 |
| **25** | My colleagues support me in implementing telemonitoring. | 41 | 5 | 2 | 7 |
| **26** | The management of my center or department supports me in implementing remote monitoring. | 41 | 5 | 3 | 7 |
| **27** | The other healthcare professionals at my center use telemonitoring in their daily practices. | 41 | 5 | 2 | 7 |
| **28** | Other healthcare professionals involved in monitoring chronic kidney disease use telemonitoring. | 41 | 5 | 2 | 5 |
| **30** | In general, healthcare professionals are well trained to use telemonitoring. | 41 | 3 | 1 | 5 |
| **31** | My daily workload has been modified by the introduction of the NeLLY service. | 22 | 7 | 5 | 8 |
| **32** | Telemonitoring has changed the way we work as a team. | 22 | 5 | 5 | 7 |
| **33** | A nursing protocol is in place for remote monitoring in my center. | 22 | 3 | 0 | 7,5 |
| **34** | I have the time I need to integrate telemonitoring into my practice (patient training and alert follow-up). | 22 | 3 | 0 | 6,75 |
| **35** | It's easy to interpret and process alerts received via the remote monitoring application. | 20 | 7 | 5 | 9 |
| **36** | Alerts are handled in collaboration between the nurse and the nephrologist. | 22 | 8 | 5 | 10 |
| **37** | Patients are motivated to use telemonitoring. | 41 | 6 | 4 | 7 |
| **38** | Patients with chronic kidney disease are sufficiently comfortable with computer tools to use telemonitoring. | 41 | 3 | 2 | 5 |
| **39** | By the time telemonitoring was implemented, I was ready to use it. | 41 | 6 | 2 | 8 |
| **40** | Today, I'm ready to use telemonitoring with my patients. | 41 | 7 | 3 | 8 |
| **41** | Today, I would recommend the use of telemonitoring to other healthcare professionals. | 22 | 7 | 4 | 10 |
| **42** | The department in which I work has the resources needed to implement remote monitoring. | 41 | 5 | 3 | 7 |
