# Supplementary material for: Barriers and Facilitators in Implementing a Telemonitoring Application for Patients With Chronic Kidney Disease and Health Professionals: Ancillary Implementation Study of the NeLLY (New Health e-Link in the Lyon Region) Stepped-Wedge Randomized Controlled Trial
Source: JMIR Mhealth Uhealth. 2025 Jan 22;13:e50014. doi: 10.2196/50014 (PMC11799818; doi:10.2196/50014)
Supplement: Multimedia Appendix 4 [file mhealth_v13i1e50014_app4.docx]

**Additional file 4.**

Table 1. Comparison of socio-demographic characteristics between respondents and non-respondents in the total source population

| **Variables** | **Non-respondent**  (N = 149)^1^ | **Respondent**  (N = 128)^1^ | **p-value**^2^ |
| --- | --- | --- | --- |
| **Gender** | | | 0.4 |
| Female | 43 (28.9%) | 43 (33.6%) |  |
| Male | 106 (71.1%) | 85 (66.4%) |  |
| **Age** | 66.99 (14.7) | 68.82 (10.1) | 0.6 |
| **Monitoring center** | | | 0.7 |
| CHU (University hospital) | 75 (50.3%) | 61 (47.7%) |  |
| Non-university hospitals | 74 (49.7%) | 67 (52.3%) |  |
| ^1^n (%); Mean (SD) | | | |
| ^2^Pearson's Chi-squared test; Wilcoxon rank sum test | | | |

Table 2. Comparison of socio-demographic characteristics between ApTelecare respondents and Mail respondant in the total source population

| **Variables** | **ApTelecare respondent**  (N = 75)^1^ | **Mail respondent**  (N = 53)^1^ | **p-value**^2^ |
| --- | --- | --- | --- |
| **Gender** | | | 0.2 |
| Female | 22 (29.3%) | 21 (39.6%) |  |
| Male | 53 (70.7%) | 32 (60.4%) |  |
| Age | 69.13 (10.6) | 68.38 (9.4) | 0.4 |
| **Monitoring center** | | | 0.088 |
| CHU (university hospital | 31 (41.3%) | 30 (56.6%) |  |
| Non-university hospital | 44 (58.7%) | 23 (43.4%) |  |
| ^1^n (%); Mean (SD) | | | |
| ^2^Pearson's Chi-squared test; Wilcoxon rank sum test | | | |

Table 3 Demographics characteristics of patients in source population and by type of user in the source population (N=277).

| **Variables** | **Total population (N=277) *** | | **Frequent user (N=65)** | | **Average user (N=85)** | | **One-off user (N=127)** | |
| --- | --- | --- | --- | --- | --- | --- | --- | --- |
|  | **N = or mean (SD)** | **%** | **N = or mean (SD)** | **%** | **N = or mean (SD)** | **%** | **N = or mean (SD)** | **%** |
| **Gender** | | | | | | | | |
| Male | 191 | 69.0 | 47 | 72,3 | 52 | 61,2 | 92 | 72,4 |
| Female | 86 | 31.0 | 18 | 27,7 | 33 | 38,8 | 35 | 27,6 |
| **Age (years)** | | | | | | | | |
| Mean (SD) | 67,8 |  | 71,0 |  | 65,9 |  | 67,5 |  |
| Min | 24 |  | 44 |  | 27 |  | 24 |  |
| Max | 95 |  | 88 |  | 89 |  | 95 |  |
| **Monitoring center** | | | | | | | | |
| CHU (university hospital) | 136 | 48,6 | 25 | 38,5 | 35 | 41,2 | 76 | 59,8 |
| Non-university hospitals/association center | 141 | 50,5 | 40 | 61,5 | 50 | 58,8 | 51 | 40,1 |
| **305 patients were initially approached, but the data description was carried out on 277 patients, i.e. approximately 9.18% missing data* | | | | | | | | |
