# Supplementary material for: Barriers and Facilitators in Implementing a Telemonitoring Application for Patients With Chronic Kidney Disease and Health Professionals: Ancillary Implementation Study of the NeLLY (New Health e-Link in the Lyon Region) Stepped-Wedge Randomized Controlled Trial
Source: JMIR Mhealth Uhealth. 2025 Jan 22;13:e50014. doi: 10.2196/50014 (PMC11799818; doi:10.2196/50014)
Supplement: Multimedia Appendix 5 [file mhealth_v13i1e50014_app5.docx]

Additional file 5

Table 1. Comparison of variables linked to technological literacy between different types of users.

| **Variables** | **Frequent user**  (N = 46)^1^ | **Average user**  (N = 39)^1^ | **One-off user**  (N = 15)^1^ | **p-value**^2^ |
| --- | --- | --- | --- | --- |
| **Help of a caregiver** | | | | 0.5 |
| Yes, always | 4 (9.30%) | 8 (22.22%) | 2 (22.22%) |  |
| Yes, sometimes | 7 (16.28%) | 7 (19.44%) | 1 (11.11%) |  |
| No | 32 (74.42%) | 21 (58.33%) | 6 (66.67%) |  |
| **Frequency of use of a computer / tablet / smartphone** | | | | 0.8 |
| Daily | 38 (82.61%) | 30 (76.92%) | 13 (86.67%) |  |
| Weekly | 3 (6.52%) | 5 (12.82%) | 1 (6.67%) |  |
| Less often | 4 (8.70%) | 2 (5.13%) | 0 (0.00%) |  |
| Never | 1 (2.17%) | 2 (5.13%) | 1 (6.67%) |  |
| **Frequency of e-mailbox consultation** | | | | 0.4 |
| Daily | 35 (76.09%) | 29 (74.36%) | 11 (73.33%) |  |
| Weekly | 4 (8.70%) | 6 (15.38%) | 4 (26.67%) |  |
| Less often | 6 (13.04%) | 2 (5.13%) | 0 (0.00%) |  |
| Never | 1 (2.17%) | 2 (5.13%) | 0 (0.00%) |  |
| **Internet connection speed** | | | | 0.7 |
| Very good | 20 (43.48%) | 13 (34.21%) | 7 (46.67%) |  |
| Pretty good | 16 (34.78%) | 13 (34.21%) | 3 (20.00%) |  |
| Medium | 8 (17.39%) | 9 (23.68%) | 3 (20.00%) |  |
| Bad | 2 (4.35%) | 3 (7.89%) | 2 (13.33%) |  |
| ^1^n (%); Mean (SD) | | | | |
| ^2^Fisher's exact test; Kruskal-Wallis rank sum test | | | | |
